# Photodynamic therapy with light-emitting diode arrays producing different light fields induces apoptosis and necrosis in gastrointestinal cancer

**DOI:** 10.3389/fonc.2022.1062666

**Published:** 2022-12-16

**Authors:** Xiafei Shi, Huijuan Yin, Xiaoxi Dong, Hongxiao Li, Yingxin Li

**Affiliations:** ^1^ Laboratory of Laser Medicine, Institute of Biomedical Engineering, Chinese Academy of Medical Sciences, Peking Union Medical College, Tianjin, China; ^2^ School of Life Sciences, Tiangong University, Tianjin, China

**Keywords:** photodynamic therapy, LED array, light field distribution, gastrointestinal cancer, apoptosis, necrosis

## Abstract

**Introduction:**

Light-emitting diodes (LEDs) have become a new light source for photodynamic therapy (PDT) because of their excellent optical properties, small size, and low cost. LED arrays have so far been designed to meet the need for accurate illumination of irregular lesions. However, LED arrays determine not only the shape of the illuminated spot but also the light field, which has a significant impact on the efficacy of PDT.

**Methods:**

We designed three types of LED arrays producing different light fields, namely an intensive LED array for a uniform light field, a sparse LED array for a non-uniform light field, and a point LED array for a Gaussian-like light field, and investigated the effect and mechanism of these light fields on PDT for gastrointestinal cancer both *in vitro* and *in vivo*.

**Results:**

We found that intensive LED-PDT induced earlier and more serious cell death, including apoptosis and necrosis, than sparse LED-PDT and point LED-PDT. Among the three LED arrays, the intensive LED array induced cells to produce more differential proteins (DEPs), mainly related to mitochondria, ribosomes, and nucleic acids. DEPs in cells subjected to sparse LED- and point LED-PDT were mainly involved in extracellular activities. For MGC-803 tumor-bearing mice, intensive LED-PDT and point LED-PDT had better tumor ablation effect than sparse LED-PDT. Notably, recurrence was observed on day 7 after sparse LED-PDT. VCAM-1 and ICAM-1 were highly expressed in sparse LEDs-PDT treated tumor tissues and were associated tumor angiogenesis, which in turn lead to poor tumor suppression.

**Conclusions:**

Therefore, the type of LED array significantly affected the performance of PDT for gastrointestinal cancer. Uniform light field with low power densities work better than non-uniform and Gaussian-like light fields.

## 1 Introduction

Gastrointestinal (GI) cancer is the fifth most common cancer worldwide and the third leading cause of cancer-related deaths according to the Global Cancer Statistics 2018 (1). There were 319,160 new diagnoses and 160,820 GI cancer-related deaths in the USA in 2018 (1, 2). At present, surgical resection is the standard treatment for resectable GI cancers, which is painful and affects postoperative quality of life. Definitive neoadjuvant chemoradiotherapy (CRT) is a treatment option for unresectable tumors and for patients who are unsuitable for or refuse surgery. However, local failure after CRT, occurring with an incidence rate of 50%–55%, is a major problem (3–5). Therefore, the exploration of new therapies for the treatment of GI cancer is important.

Developed in recent decades, photodynamic therapy (PDT) is a cancer treatment modality. In PDT, photosensitizers are first injected into patients or used to cover the surface of the skin for a certain time to selectively combine with tumor tissues, and then light of a specific wavelength is used to initiate a photochemical reaction with tissue oxygen to generate reactive oxygen species (ROS), which acts to kill cancer cells (6, 7). PDT is now widely used in the clinic to treat tumors owing to its multiple advantages, such as non-invasiveness, high tumor selectivity, good cosmetic effect with small or no scarring, low tumor recurrence, and low cross-resistance (8, 9). In the treatment of GI cancer, the cavity structure of the GI tract makes it feasible to perform PDT because light can be introduced to the GI tract lesion through the optical fiber of an endoscope (10, 11). Although treatment efficacy is remarkable, it is highly dependent on endoscopy and therefore has the same shortcomings as endoscopy, including high cost and complicated operation procedures.

Fortunately, existing photosensitizers can be initiated with relatively low-cost light-emitting diodes (LEDs), which have been proven to be as effective as traditional medical lasers (12–18). Moreover, LEDs can be powered by batteries or charged wirelessly, making them highly mobile, easy to carry, and easy to operate (17). In addition, therapy can be guided and monitored using photosensitizer fluorescence imaging with consumer smartphones, which also offers the potential for telemedicine integration (16, 18). Accompanied by easy-to-use light sources with calibrated dosimetry, PDT can become an effective treatment modality for global health settings. However, parameters such as wavelength, luminous flux, luminous intensity, luminous efficiency, and light intensity distribution, namely light field, affect the therapeutic efficacy of LED-PDT. For practical applications, LED arrays are designed and manufactured according to specific illumination requirements. Hadis et al. (19) demonstrated that an LED array comprising 96-well plates could produce wavelengths (400–850 nm) that were effective against cells *in vivo* at high irradiances (48–142 mW/cm^2^). Jamali et al. (20) developed a light source producing two wavelengths (red and blue) for each well of a 96-well plate and found that the light power densities required for PDT of human glioma cell lines was 50 μW/cm^2^ and 25 μW/cm^2^ for the blue and red LED, respectively. Yamagishi et al. (21) developed an implantable and wirelessly powered PDT device consisting of LED chips and bioadhesive and stretchable polydopamine-modified poly(dimethylsiloxane) nanosheets and, furthermore, confirmed that a fluence rate of 33 μW/cm^2^ for red LED devices and 75 μW/cm^2^ for green LED devices had a good tumor ablation effect. Because standard LED equipment is unavailable on the market for research and treatment, there is a lack of accepted standards for LED irradiation parameters with corresponding PDT effects.

Light, photosensitizer, and oxygen molecules are the three major elements of PDT (22–24). Therefore, oxygen distribution in cells or tissues should be fully considered in the design of light sources and choice of photosensitizers. De Souza et al. (25) showed that oxygen consumed by tissues was restored during the interval and that the efficiency of PDT was improved if fractioned light is used. In particular, by using light/dark interval lighting, tissues receiving low-intensity light may transport oxygen to surrounding tissues in which oxygen consumption increases rapidly owing to high-intensity light irradiation. Therefore, by controlling the light/dark cycle, tissue oxygen reperfusion can be promoted to compensate for oxygen consumption during the photochemical reaction, which may improve the efficiency of PDT (26–28). Similarly, owing to oxygen fluidity in the tissue, different light fields (such as uniform, non-uniform, and Gaussian-like light fields) lead to different oxygen utilization rates in the irradiation area, which may affect the PDT effect.

Therefore, we designed three LED arrays producing different light fields and investigated the effect and mechanism of these light fields on PDT for GI cancer, with the aim of seeking the best LED light field distribution, and provide reference and basis for preparing small LED light sources, such as PDT capsules, for digestive tract cancer in future, hoping further promote the clinical application of PDT in the treatment of digestive tract tumor.

## 2 Materials and methods

### 2.1 Preparation and characterization of LED arrays with different light fields

Three LED arrays with different light fields were constructed: an intensive LED array to produce a uniform light field, a sparse LED array to produce a non-uniform light field, and a point LED array to produce a Gaussian-like light field. Algorithmic analysis was used to define each light field, as shown in the **Figure S1**. From this analysis, light sources for cell and animal experiments were designed and manufactured.

For *in vitro* cell experiments, a module corresponding to 6-well plates was designed for the different LED arrays, as shown in **Figure S2 (A)**. The module consisted of 208 LEDs, 16 LEDs, and 1 LED for the intensive LED array, sparse LED array, and point LED array, respectively.

For *in vivo* animal experiments, light sources with different light fields were used, as shown in **Figure S2 (B)**. For the intensive LED array, 25 LEDs were closely arranged to form a 5 × 5 array, while for the sparse LED array, 4 LEDs were uniformly arranged at intervals of 0.4 cm to form a 2 × 2 array, and for the point LED array, 1 LED was placed in the center position.

Moreover, heat generation was a prerequisite of LEDs and a specific heat dissipation design using silica gel sheets with good thermal conductivity was made, thus the thermal damage of LED to cells was successfully avoided, as shown in **Figure S3**.

### 2.2 Cell culture

Human gastric cancer cell NCI-N87 (Highly differentiated) and HGC-27 (Undifferentiated), and Human colorectal adenocarcinoma epithelial cell SW837, purchased from National Infrastructure of Cell Line Resource (Beijing, China), were maintained in RPMI 1640 medium (Gibco, American) supplemented with 10% fetal bovine serum (FBS) and 1% penicillin and streptomycin. Human gastric cancer cell MGC-803 (Poorly differentiated) and Human colon cancer cell HT-29, obtained from the Institute of Biochemistry and Cell Biology, Chinese Academy of Sciences (Shanghai, China), were cultured in Dulbecco’s modified Eagle’s medium (DMEM) (Gibco) and DMEM/F12 medium (Gibco), respectively. The media were supplemented with 10% FBS and 1% penicillin (Gibco). All cells were cultured at 37 °C in 5% CO_2_ and tested negative for mycoplasma contamination.

### 2.3 Optimization of PDT dose in GI cancer cells

PDT experiments were conducted using hematoporphyrin derivatives (HpD) and the NCI-N87, MGC-803, HGC-27, HT-29, and SW837 cell lines. Cells were plated into sterile 96-well plates. All cell lines (2–5×10^4^ cells/mL) were separately incubated for 4 h with the photosensitizer at various concentrations (3.125, 6.25, 12.5, 25, 50 μg/mL), and each mixture was exposed to light of various energy densities (0, 3, 6, 12, 24 J/cm^2^) and power densities (1.25, 2.5, 5.0, 10, 20 mW/cm^2^). The control experimental conditions were (1) no HpD and no light, (2) HpD and no light, and (3) light and no HpD. Each condition was repeated in triplicate. A custom-designed LED light source (635 ± 15 nm) was used. The MTT assay was used to determine cell activity at 24 h after PDT. The optimal PDT effect dose for each cell line was selected.

### 2.4 LED-PDT effect detected by live/dead cell staining

Cells (2×10^5^ cells/sample) were seeded on the glass slide within 6-well plates and then subjected to PDT with three different LED arrays at the selected PDT doses. At 24 h after PDT, the cells were double-stained by adding 210 μL of phosphate-buffered saline (PBS) containing Calcein AM (2 μM) and Ethidium homodimer-1 (EthD-1, 4μM) and then incubated at room temperature for 40 min in the dark at 37 °C. The cells were scanned (20× magnification) using a digital pathology scanner (NanoZoomer S60, Hamamatsu, Japan). Green Calcein AM fluorescence and red EthD-1 fluorescence were detected on the FITC and TxRED channels, respectively. Cell survival was analyzed using ImageJ.

### 2.5 Apoptosis assays

At 12 h after PDT with the three LED arrays, the cells were collected by trypsinization, centrifuged at 1000 × g for 5 min, washed once with cold PBS, and then resuspended in 195 μL of cold binding buffer containing annexin V-FITC (5 μL) and propidium iodide (PI, 10 μL) following the manufacturer’s instructions. Finally, the cells were analyzed using a FACSAria flow cytometer (BD Biosciences). Green FITC fluorescence and red PI fluorescence were measured at 515–545 nm and 565–606 nm, respectively.

### 2.6 ELISA for ATP and iNOS

At 6 h after PDT, cells were carefully washed with cold PBS, and 200 μL of protein extraction buffer provided in the ELISA kit (Beyotime, China) was then added to each well. The cells were incubated on ice for 10 min to ensure that the cells were fully lysed and the proteins were released. The protein concentration was measured with a BCA protein concentration kit (Beyotime, China). ATP and iNOS concentrations were separately determined using ELISA kits following the manufacturer’s suggested protocol.

### 2.7 Proteomics study

NCI-N87 and MGC-803 cells were subjected to PDT with the intensive LED, sparse LED, and point LED arrays. Cell samples were collected at 6 h after PDT. At least six biological replicates were obtained for each case. Extracted proteins were analyzed using liquid chromatography–tandem mass spectrometry (LC–MS/MS) combined with tandem mass tag (TMT) labeling. Briefly, pooled proteins from eight groups, MGC-C, MGC-1, MGC-2, MGC-3, N87-C, N87-1, N87-2, and N87-3, were cleaved into peptides using 1 μg/μL trypsin and isobaric labeled TMT. Equal protein amounts derived from each group were labeled with different TMT labels: MGC-C, TMT-127_N; MGC-1, TMT-127_C; MGC-2, TMT-128_N; MGC-3, TMT-128_C; N87-C, TMT-129_N; N87-1; TMT-129_C; N87-2, TMT-130_N; and N87-3, TMT-130_C. Following mixing, drying, and fractioning into 10 fractions by high-performance liquid chromatography (HPLC), mixtures of the labeled peptides were loaded onto a reversed-phase C18 column (75 μm × 2 cm, 3 μm, 100 Å; Acclaim PePmap, Thermo Scientific) and separated with a reversed-phase C18 column (75 μm × 10 cm, 5 μm, 300 Å; Agela Technologies) mounted on a nano-LC system (Dionex Ultimate 3000). Proteins were characterized using a mass spectrometer (Q-Exactive, Thermo Fisher Scientific, MA, USA).

For data analysis, a false discovery rate (FDR) of less than 1.0% with a confidence level of 95% was selected for identification. Accurately quantified protein was expressed as the ratio of protein between samples. The threshold of upregulation was defined as 1.2, while that for downregulation was 0.8. Gene Ontology (GO) annotation and the Kyoto Encyclopedia of Genes and Genomes (KEGG) were employed to determine functional classification and the significant pathways of DEPs, respectively.

### 2.8 Animal model

Animal experiments were approved by the Tianjin Animal Ethics Committee (SYXK(JIN):2019-0002). Nine-week-old BALB/c nude mice were obtained from Beijing Vital River Laboratory Technology Co. Cultured monolayers of MGC-803 cells were collected and resuspended in PBS, of which 0.2 mL (4 × 10^6^ cells) were subcutaneously injected into the right back buttocks of the mice. After cell seeding, the animals were maintained under standard conditions for approximately 2 weeks until the tumor grew to 0.8 cm in diameter.

### 2.9 *In vivo* PDT with LED arrays

We used 80 MGC-803 tumor-bearing mice, which were equally divided into control and different PDT groups, as shown in **Table 1**. HpD (H20064266, 5 mL:25 mg per ampoule) was injected into the tail vein of the mice at 10 mg/kg. After 24 h in the dark, mice were exposed to intensive LED-PDT, sparse LED-PDT, and point LED-PDT. After PDT, mice were maintained under standard conditions in the dark and observed every other day. The tumor size was measured using a vernier caliper, and the tumor volume was calculated according to the following formula: V_t_=1/2×a×b^2^, where a is the longer diameter, b is the shorter diameter, and t is the number of days after PDT.

**Table 1:** Groups of tumor-bearing mice and corresponding PDT parameters.

Tumor-bearing mice		Groups		PDT parameters			HpD (mg/kg)
				Power density (mW/cm^2^)	Irradiation time (min)	Energy density (J/cm^2^)	
MGC-803	1	Control		—			10
	2	Intensive LED-PDT	Low	20	20	24	
	3		Middle		40	48	
	4		High		80	96	
	5	Sparse LED-PDT	Low		20	24	
	6		Middle		40	48	
	7		High		80	96	
	8	Point LED-PDT	Low		20	24	
	9		Middle		40	48	
	10		High		80	96	

### 2.10 H&E staining and immunohistochemistry

The mice were sacrificed at 3, 7, and 14 days after PDT by euthanasia, and the tumor tissue was harvested, fixed with 4% paraformaldehyde, embedded in paraffin, and sectioned into slices of 5 μm in thickness. Standard H&E staining (Solarbio, Beijing, China) was conducted to visualize necrotic regions. A Click-iT TUNEL Colorimetric IHC Detection Kit (C10625, Thermo Fisher, USA) was used to detect apoptosis. Moreover, Ki67, vascular cell adhesion molecule-1 (VCAM-1), and intercellular adhesion molecular-1 (ICAM-1) indicators were used to test for tumor proliferation and recurrence. Sections were imaged (20× magnification) using a digital pathology scanner (NanoZoomer S60, Hamamatsu, Japan). Positive cells were quantitatively assessed using ImageJ.

### 2.11 Statistical analysis

All data were presented as the mean ± standard deviation (SD). Comparisons between groups were performed using one-way ANOVA. Significance was set at p<0.05. Statistical analyses were performed using SPSS v19.0 (SPSS Statistics).

## 3 Results

### 3.1 Light fields of LED arrays

**Figure 1:**
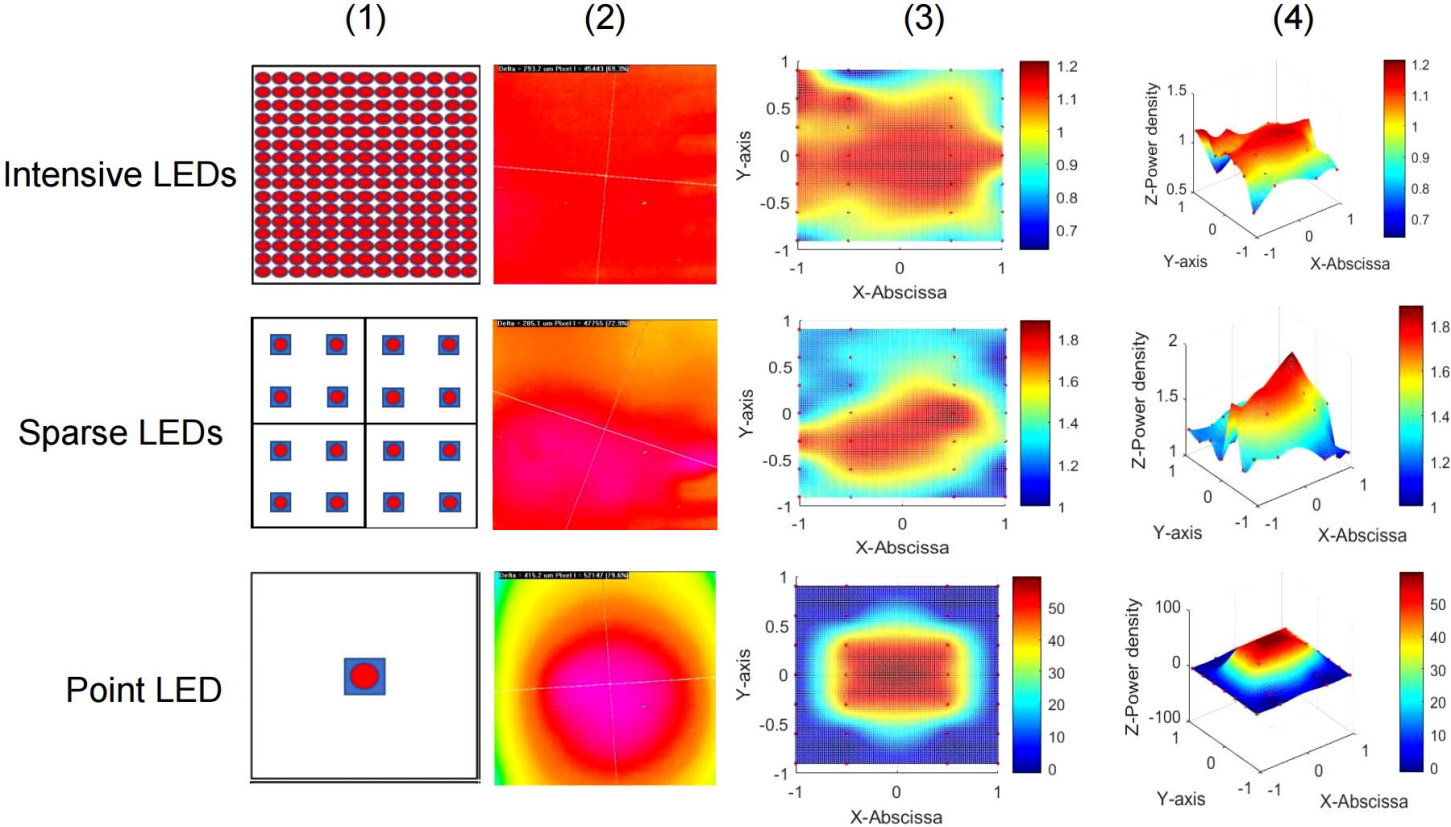
Three LED arrays with different light fields for *in vitro* experiments. Top: Intensive LED array producing a uniform light field. Middle: Sparse LED array producing a non-uniform light field. Bottom: Point LED array producing a Gaussian-like light field. Performance of the three LED arrays: (1) Schematic diagram, (2) light spot, (3) two-dimensional heat map of power density distribution, and (4) three-dimensional heat map of power density distribution.

To investigate the influence of LED light fields on the PDT of GI cancer, we designed three different LED arrays for *in vitro* experiments. The intensive LED array, producing a uniform light field, consisted of 208 LEDs closely arranged in a 13 × 6 array. The sparse LED array, producing a non-uniform light field, was composed of 4 arrays (2 × 2), including 16 LEDs, with a center distance of 9 mm between two adjacent LEDs. The point LED array, approximating a Gaussian-like light field, contained only one LED placed in the center. **Figure 1** shows diagrams of the above-mentioned LED arrays, their light spots, and power density distribution in two and three dimensions. And the central wavelength of three LED arrays was about 635 nm with bandwidth being 15 nm, as shown in **Figure S4**.

The Monte Carlo method was used to simulate propagation of the light field produced by the three LED arrays designed for *in vivo* experiments. As shown in **Figure S5**, in tissue, the transmission distance of light from the intensive LED and sparse LED arrays was 6000 mm, which was significantly deeper than that of light from the point LED array (4000 mm). However, at each tissue depth, the absorption and luminous flux of light from the sparse LED array were the lowest among light from the three LED arrays.

### 3.2 Effects of three LED arrays on PDT of GI cancer

We first analyzed the performance of LED-PDT using a series of doses and five types of GI cancer cells (NCI-N87, MGC-803, HGC-27, SW837, and HT-29) to select the optimal dose for a comparison of the LED arrays with different light fields. As shown in **Figure S6**, the five GI cancer cell lines have different sensitivities toward PDT. The tumor-suppression effect of PDT was correlated with cell type, tumor differentiation degree, and PDT dose. The optimal PDT parameters (minimum dose with cell mortality greater than 70%) for each cell line were obtained, as shown in **Table S2**.

As shown in **Figure S7(A)**, the cell viability was above 90% when exposed only to light from LED array at different energy density (0, 3, 6, 12 and 24 J/cm^2^), which was almost consistent with the no HpD and no light group. While MGC-803 and HGC-27 cells showed slight dark toxicity as the concentration of HpD increasing gradually (**Figure S7B**). Therefore, we took the HpD and no light group as control group to explore the effect and mechanism of PDT mediated by three different LED arrays in the follow-up experiments, ensureing that the differences in cell death were entirely induced by different light fields.

**Figure 2:**
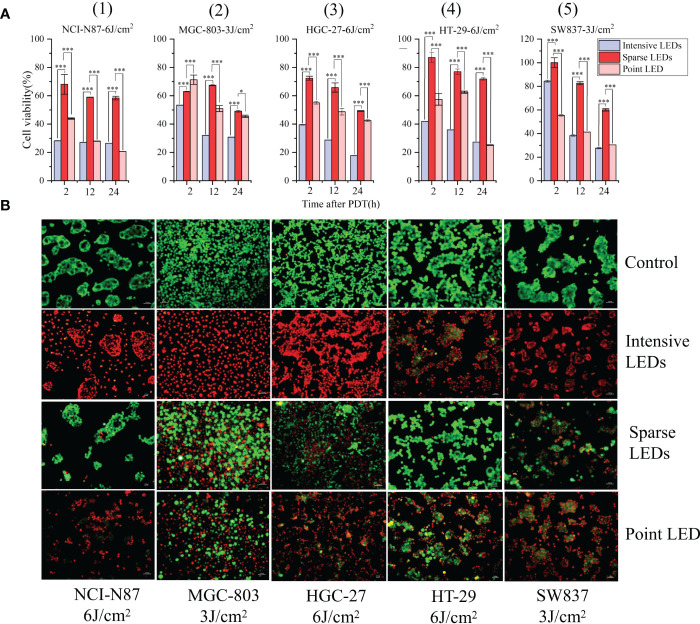
Effect of different light fields on the performance of PDT for GI cancer. **(A)** Viability of five different GI cancer cells after PDT mediated by different light fields: (1) NCI-N87, (2) MGC-803, (3) HGC-27, (4) HT-29, and (5) SW837. **(B)** Differences in cell viability after irradiation with no light (control), uniform light field (intensive LED array), non-uniform light field (sparse LED array), and Gaussian-like light field (point LED array). Cells were stained with Calcein AM/EthD-1 at 24 h post-PDT. Calcein AM (green) and EthD-1 (red) indicate living and dead cells, respectively. (*P < 0.05, and ***P < 0.001 by one-way ANOVA. Mean ± SD in bar graphs.).

The viability of NCI-N87, MGC-803, HGC-27, SW837 and HT-29 cells were determined using the MTT assay at 24 h after PDT with the three LED arrays, and the resulting temporal dynamics of PDT-mediated cytotoxicity was compared. The intensive LED and point LED arrays caused earlier and more severe cell damage than the sparse LED array. As shown in **Figure 2A**(1), the survival rates of NCI-N87 cells decreased to 28.24%, 43.58%, and 68.10% at 2 h after intensive LED-PDT, point LED-PDT, and sparse LED-PDT, respectively. Additionally, the survival rates of NCI-N87 cells decreased to 26.41%, 20.66%, and 58.39% at 24 h after PDT with the three LED arrays. Remarkably, at 2 h post-treatment, cell damaged by intensive LED-PDT was 2.25 times and 1.28 times greater than that by sparse LED-PDT and point LED-PDT, respectively (**Figure 2A**(1)). Similar results were obtained for MGC-803, HGC-27, HT-29, and SW837 cell lines, as shown in **Figure 2A**(2)–(5).

To observe the effect of light intensity heterogeneity in the irradiation region on cell damage, cells were stained with Calcein AM/EthD-1. Cells were identified as living (Calcein AM, green) or dead (EthD-1, red) depending on the observed fluorescence signals. As shown in **Figure 2B**, at the same dose, the intensive LED array resulted in homogenous cell death, while the sparse LED array significantly enhanced cell damage, and no regional characteristics corresponding to light intensity were found. The point LED array resulted in cell death in the central region (with strong light intensity). By counting the dead cells, mortality caused by the three LED arrays was found to be consistent with the above MTT results.

### 3.3 PDT-induced apoptosis mediated by different light fields

**Figure 3:**
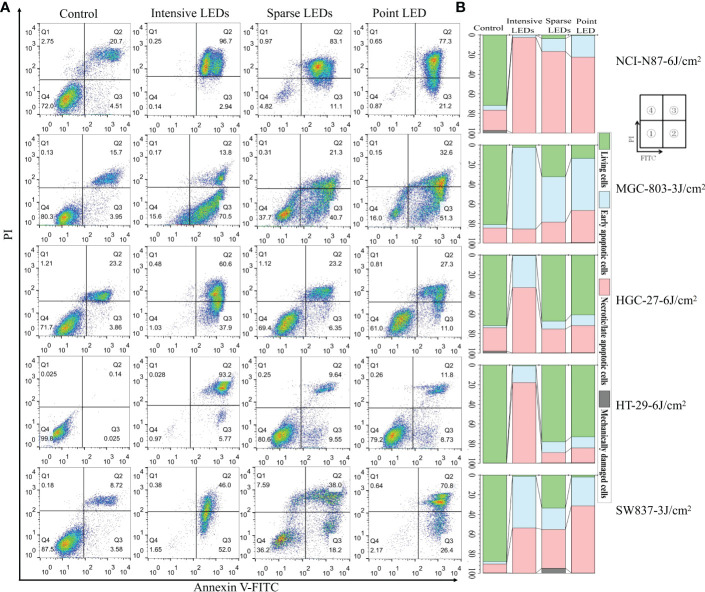
Apoptosis of GI cancer cells induced by PDT with different light fields. Cells were first irradiated with the intensive LED, sparse LED, and point LED arrays, and then the apoptosis were detected. **(A)** Apoptosis rate: Cells were stained with annexin-FITC/PI at 12 h post-PDT. Annexin V-FITC and PI stains indicate apoptotic and necrotic cells, respectively. ① Living cells, ② early apoptotic cells, ③ necrotic/late apoptotic cells, and ④ mechanically damaged cells. **(B)** Percentage stacked bar chart corresponding to **(A)**.

To quantify apoptosis, cells were first double-stained with Annexin V-FITC/PI and then analyzed using flow cytometry (FCM). Cells were classified as living (FITC−/PI−), early apoptotic (FITC+/PI−), late apoptotic/necrotic (FITC+/PI+), and mechanically damaged (FITC−/PI+). As shown in **Figures 3A, B**, intensive LED-PDT caused more apoptotic or necrotic cells than sparse LED-PDT and point LED-PDT, especially for HGC-27 and HT-29 cell lines. The rates of early apoptosis and late apoptosis/necrosis for HGC-27 cells were respectively 32.8% and 66.7% (total of 99.5%) after intensive LED-PDT, 8.08% and 24.3% after sparse LED-PDT, and 11.1% and 27.5% after point LED-PDT. HT-29 cells were significantly more sensitive to intensive LED-PDT, with a total apoptosis rate of 99.26%, than sparse LED-PDT and point LED-PDT, with a total apoptosis rate of 21.9% and 26.8%, respectively.

**Figure 4:**
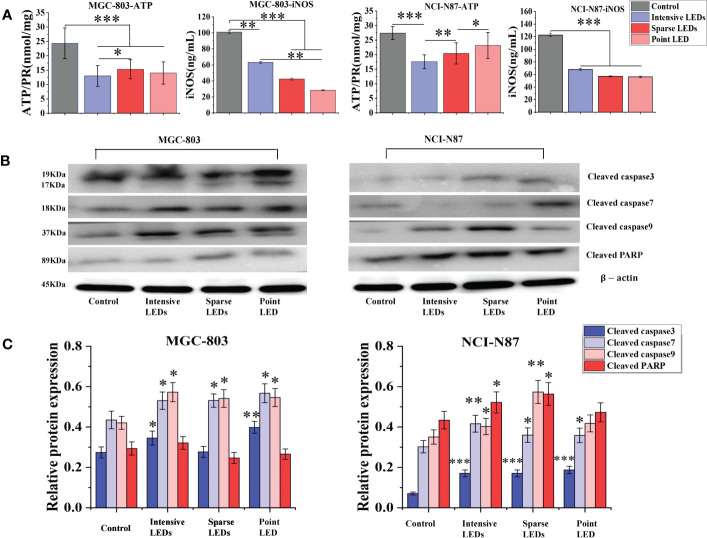
Apoptosis of GI cancer cells induced by PDT with different light fields. Cells were first irradiated with the intensive LED, sparse LED, and point LED arrays, and then the apoptosis-related factors and apoptosis-related proteins were detected. **(A)** Apoptosis-related factors: Cells were collected, and the content of ATP and iNOS were quantified following the kit instructions. **(B)** Apoptosis-related proteins: Western blotting analysis was used to evaluate the cleaved products of apoptosis-related proteins (cleaved caspase3, cleaved caspase7, cleaved caspase9, and cleaved PARP) in cell samples at 6 h after PDT, and the relative protein expression was quantified using ImageJ **(C)**. (*P < 0.05, **P < 0.01, ***P < 0.001 by one-way ANOVA. Mean ± SD in bar graphs.).

To further compare apoptosis induced by the three LED arrays, ATP and iNOS related to the mitochondrial apoptosis pathway were detected (29–33). Groups subjected to PDT with three LED arrays had significantly reduced intracellular ATP levels than the control group (**Figure 4A**), and the intensive LED array had the strongest effect among the three LED arrays (p<0.05). For NCI-N87 cells, the ATP levels were 17.59 ± 2.37, 20.42 ± 3.64, and 23.18 ± 4.42 mol/mg after intensive LED-PDT, sparse LED-PDT, and point LED-PDT, respectively. The iNOS levels of the NCI-N87 and MGC-803 cells were also significantly decreased after PDT with the three LED arrays compared with that of the control group (**Figure 4A**).

We also evaluated the cleaved products of caspase3, caspase7, caspase9, and PARP, key proteins in the mitochondrial apoptosis pathway, using western blotting. As shown in **Figure 4B**, the expressions of cleaved caspase3, cleaved caspase7, and cleaved caspase9 in MGC-803 cells were all upregulated after PDT with the three LED arrays compared with that of the control. Cleaved caspase3 expression had the most significant difference among the three LED array groups, which was 1.30 times and 1.44 times higher in the intensive LED and point LED groups than in the sparse LED group, respectively (**Figure 4C**). Moreover, the upregulation of cleaved caspase9 and cleaved PARP after intensive LED-PDT was the most obvious compared with the other two groups. Especially, the relative protein expression of cleaved caspase9 was 0.57 ± 0.04 in the intensive LED-PDT group, which was 1.06 and 1.05 times higher than in the sparse LED and point LED groups, respectively (**Figure 4C**).

For NCI-N87 cells, although the expression of cleaved caspase3 protein in the three LED array groups were significantly upregulated compared with that of the control group, there was no significant difference among the three groups. However, there were significant differences in the expression of cleaved caspase9 among the three groups, which was significantly higher in the sparse LED-PDT group (0.57 ± 0.05) than in the other two groups (intensive LED-PDT: 0.40 ± 0.04; point LED-PDT: 0.41 ± 0.04) (**Figure 4C**).

### 3.4 Proteomics changes caused by PDT with different light fields

**Figure 5:**
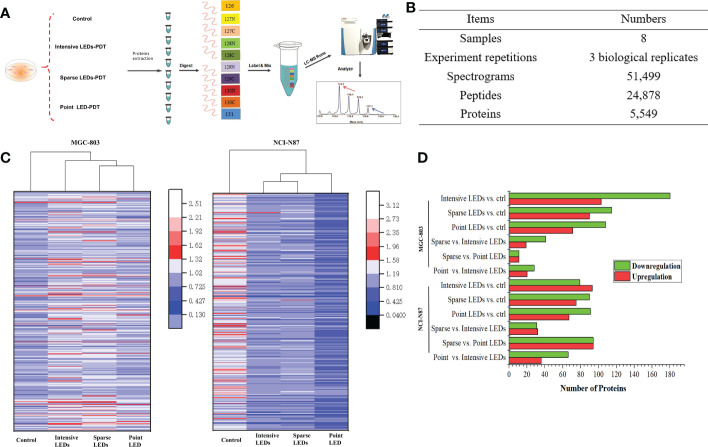
Proteomic analysis of GI cancer cells treated with intensive LED-PDT, sparse LED-PDT, and point LED-PDT, respectively. **(A)** The experimental procedure. **(B)** General information of the proteomic analysis. **(C)** Heat map analysis of 500 proteins with the most significantly altered expression levels. The heat map was produced using Origin. **(D)** Distributions of DEPs of paired comparisons among intensive LED-PDT, sparse LED-PDT, point LED-PDT, and control (ctrl) groups. Red and green represent upregulated and downregulated DEPs, respectively.

A tandem mass tags labelling combined liquid chromatography-tandem mass spectrometry (TMT-MS/MS) analysis was performed to determine the proteomics of the MGC-803 and HGC-27 cells among the PDT groups according to the experimental procedure shown in the **Figure 5A**. Of the 24,878 peptides, 5549 proteins were identified, which matched the 51,499 MS/MS spectra at a false discovery rate (FDR) of 1% (**Figure 5B**). The differentially expressed proteins (DEPs) of paired comparisons among the three LED array PDT groups and control groups were determined. We selected the most significant 500 DEPs (p<0.05) in the four groups by paired comparisons for overall analysis of changes in protein expression trends using heat map clustering, as shown in **Figure 5C**. These results demonstrated that expression of proteins after intensive LED-PDT, sparse LED-PDT, and point LED-PDT were significantly different, especially for MGC-803 cells.

To identify DEPs within the obtained human protein dataset, we calculated the number of folds changed identified proteins with significantly (P<0.05) increased (1.2-fold) or decreased (0.83-fold) levels of accumulation. Taking MGC-803 cells as an example, 283, 205, 179, 60, 22, and 48 DEPs were identified in paired comparisons of intensive LED-PDT versus control, sparse LED-PDT versus control, point LED-PDT versus control, sparse LED-PDT versus intensive LED-PDT, sparse LED-PDT versus point LED-PDT, and point LED-PDT versus intensive LEDs-PDT, respectively (**Figure 5D**).

**Figure 6:**
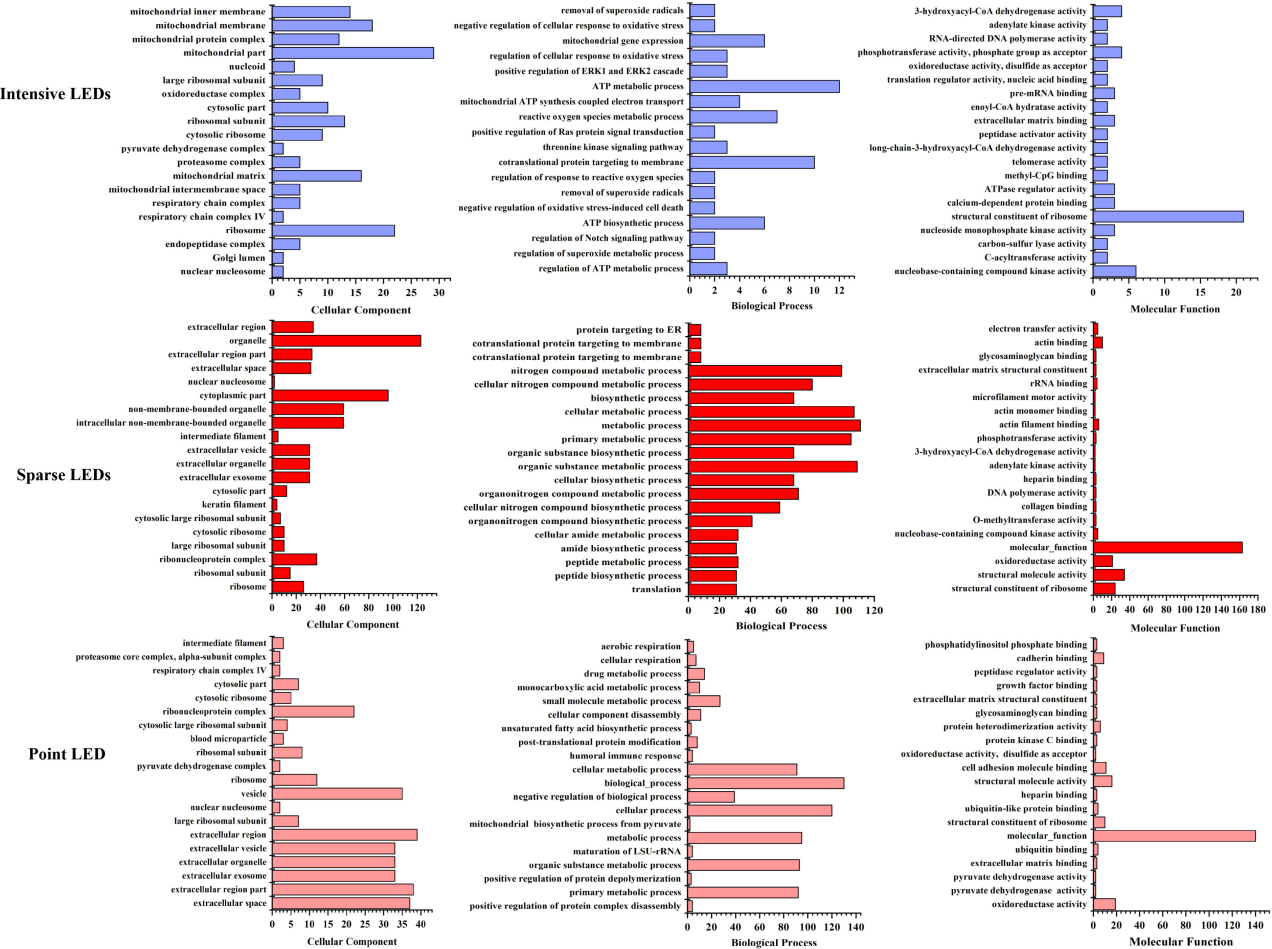
Assessment of the 20 most significant DEPs with the smallest p-value after intensive LED-PDT (top), sparse LED-PDT (middle), and point LED-PDT (bottom) using Gene Ontology (GO). DEPs were analyzed according to cellular component (left), biological process (middle), and molecular function (right).

Gene Ontology (GO) analysis was used to obtain the functional annotation information of the DEPs, including cellular components, biological processes, and molecular functions. The top 20 DEPs with the smallest *p*-values were mapped, as shown in **Figure 6**. Compared with the control group, DEPs induced by intensive LED-PDT were mainly related to the structure and function of mitochondria and ribosomes, of which 12 DEPs were involved in ATP synthesis and metabolism. Sparse LED-PDT and point LED-PDT induced similar DEPs, mainly involved in extracellular activities and ribosome function. Therefore, PDT with different light fields may give rise to different mechanisms of cell death.

**Figure 7:**
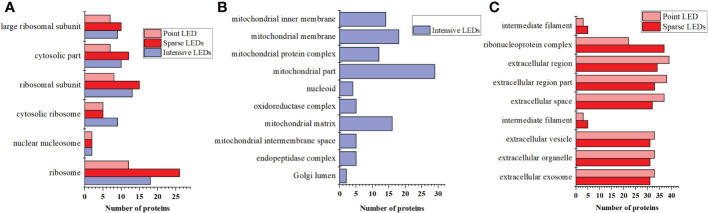
DEPs that were the same and different after three types of PDT: **(A)** The same DEPs after three types of PDT. **(B)** The unique DEPs induced by intensive LED-PDT. **(C)** The same DEPs after sparse LED-PDT and point LED-PDT.

To further analyze the mechanism of cell death caused by PDT with different light fields, we identified the DEPs that were the same and different among the three LED groups and analyzed the biological processes and molecular functions of these DEPs. As shown in **Figure 7A**, DEPs that were the same among the three LED groups were mainly from six cellular components: ribosome, nuclear nucleosome, cytosolic ribosome, ribosomal subunit, cytosolic part, and large ribosomal subunit. Among them, ribosomes produced the most DEPs after intensive LED-PDT, sparse LED-PDT, and point LED-PDT (18, 26 and 12, respectively).

The intensive LED array group had 9 DEPs that were different from those of the other two groups. These 9 DEPs were mainly related to mitochondrial components, such as mitochondrial part, mitochondrial membrane, mitochondrial matrix, mitochondrial inner membrane, and mitochondrial protein complex (**Figure 7B**). The sparse LED array and point LED groups also had 9 DEPs that were different from those of the intensive LED group. These 9 DEPs were mainly involved in the cell components of the ribonucleoprotein complex, extracellular region, extracellular space, extracellular exosome, extracellular organelle, and extracellular vesicle (**Figure 7C**).

### 3.5 LEDs-PDT induced tumor inhibition in mice

Before comparing the effects of different light fields on the performance of PDT, we determined the appropriate light dose and tumor-bearing mouse model. For the former, we used the intensive LED array to irradiate tumor-bearing (MGC-803 cells) mice at a power density of 20 mW/cm^2^ for 20, 40, and 80 min, corresponding to energy densities of 24, 48, and 96 J/cm^2^, respectively. As shown in **Figure S8**, good tumor ablation occurred with doses of 48 and 96 J/cm^2^, and after 4 days, the tumor volume decreased significantly, by 76.47% and 49.59%, respectively. There was no tumor recurrence nor metastasis after 15 days. However, side effects, namely large areas of black scabs, appeared after irradiation with a dose of 96 J/cm^2^. The necrotic scab was small and completely subsided 14 days after irradiation with a dose of 48 J/cm^2^. However, irradiation with a dose of 24 J/cm^2^ did not lead to necrotic scabs and could not effectively inhibit tumor growth. Therefore, 48 J/cm^2^ was selected as the optimal dose for subsequent experiments.

**Figure 8:**
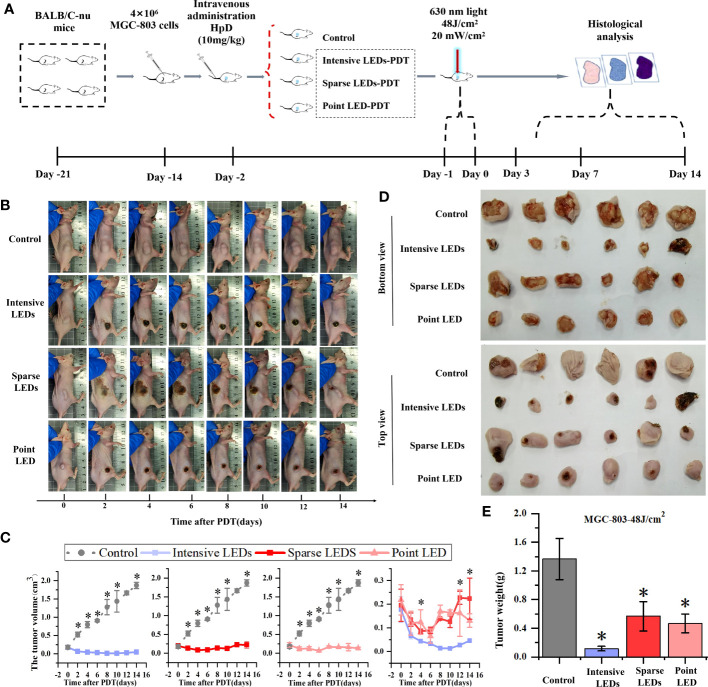
Effect of PDT mediated by different light fields on MGC-803 tumor-bearing mice. **(A)** Timeline of the PDT procedure. **(B)** Photographs of the mice at different days after intensive LED-PDT, sparse LED-PDT, and point LED-PDT. **(C)** Evolution of tumor volumes after PDT. **(D)** Photographs of resected tumors 14 days after PDT. € Volumes of resected tumors 14 days after PDT. (Mean ± SD, *P < 0.05 by one-way ANOVA, n = 3 mice for **(C)**, n = 6 mice for **(E)**.

MGC-803 tumor-bearing mice were subjected to PDT with different light fields at the dose of 48J/cm^2^, and the results were compared accorrding to the experimental procedure shown in **Figure 8A**. On day 2 after intensive LED-PDT, tumor necrosis occurred and the tumor volume decreased from 0.18 ± 0.05 cm^3^ to 0.06 ± 0.001 cm^3^. By day 14 (observation end point), the tumor volume (scab) decreased to 0.027 ± 0.004 cm^3^, which was considered complete disappearance of the tumor (**Figures 8B, C**). The necrotic scar was small throughout the observation period. After sparse LED-PDT, the necrotic scar was very large, occupying half of the back, and gradually shrank until day 6. However, from day 8, the tumor reappeared in the place where the scar disappeared, and by day 14, the recurrent tumor increased to its original size. After point LED-PDT, tumors showed signs of necrosis, were covered with scabs, and gradually shrank but recurred around the scabs on day 8. At the end of the observation period (day 14), tumor tissues of mice were collected and weighed, as shown in **Figures 8D, E**. The average weights of tumors subjected to intensive LED-PDT, sparse LED-PDT, and point LED-PDT were respectively 0.12 ± 0.03 g, 0.57 ± 0.20 g, and 0.46 ± 0.13 g, which were significantly lower than that (1.36 ± 0.28 g) of tumors in the control group (P<0.05).

### 3.6 Histological pathology induced by LEDs-PDT

**Figure 9:**
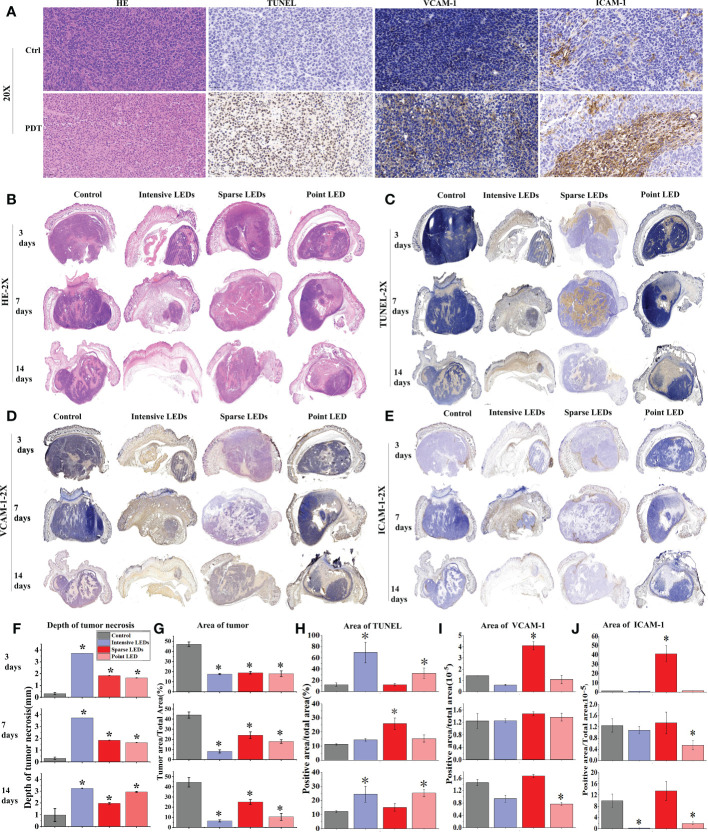
MGC-803 tumor cell necrosis, apoptosis, and angiogenesis after PDT mediated by different light fields. MGC-803 tumor-bearing mice were sacrificed on day 3, 7, and 14 after PDT. **(A)** Tumor tissue sections of control and intensive LED-PDT groups were stained with HE, TUNEL, VCAM-1, and ICAM-1 and then scanned with a 40× microscope, and images were magnified 20×. Coronal section scans of tumor tissue stained with different indicators: **(B)** HE, **(C)** TUNEL, **(D)** VCAM-1, and **(E)** ICAM-1. Quantitative analysis of aforementioned different indicators staining results: **(F)** depth of tumor necrosis and **(G)** area of residual tumor from HE staining. **(H)** Area of positive cells in TUNEL staining, representing tumor apoptosis. (**I, J)** Area of positive cells in VCAM-1 **(I)** and ICAM-1 **(J)** staining, positively correlated with tumor angiogenesis. (Mean ± SD, *P < 0.05 by one-way ANOVA compared with control group).

Histopathological staining (HE, TUNEL, Ki-67, VCAM-1, and ICAM-1) was used to determine the mechanism of tumor suppression caused by PDT with different light fields. Staining with HE and TUNEL revealed that PDT caused tumor necrosis and apoptosis, respectively (**Figure 9A**). We described the results of intensive LED-PDT as an example. In HE staining, compared with the dense nests of tumor cells in the control group (dark blue staining), tumor cells were significantly necrotic (pink staining, no cell structure) after PDT. Staining with TUNEL revealed that PDT resulted in a large number of brown stained apoptotic cells. Ki-67 labeling was used to analyze cell proliferation, while VCAM-1 and ICAM-1 staining was used to reflect angiogenesis. These three indicators were mainly observed in relation to tumor recurrence in this study.

Coronal section scans of tumor tissue were used to demonstrate the efficacy of PDT. Changes in the HE, TUNEL, VCAM-1, and ICAM-1 indicators were recorded at different observation time points (3, 7, and 14 days) after PDT with different LED arrays (**Figures 9B–E**), and the corresponding quantified changes were plotted in bar charts (**Figures 9F–J**). The size of necrotic tumors gradually increased with time, although there were significant differences under different light fields. As shown in **Figures 9B, F, G**, intensive LED-PDT resulted in large and deep necrotic tumors (3725.48 ± 0.81 μm on day 3). After sparse LED-PDT, tumor necrosis was incomplete and tumor recurrence was observed on day 14. Although point LED-PDT led to shallow necrotic tumors (1637.94 ± 22.83 μm on day 3, 2044.93 ± 59.68 μm on day 7, and 2938.16 ± 45.48 μm on day 14), tumor necrosis was complete within the irradiation area and recurrence of only deep residual tumors was observed. These results were consistent with the Monte Carlo simulations of the penetration depths of light from different LED arrays in tumor tissues (**Figure S3**).

Apoptosis and inflammation are the main secondary effects of PDT. From the results of TUNEL staining, apoptotic cells accounted for a large proportion of residual tumor nests after intensive LED-PDT (69.39% on day 3), and the residual tumor nests were found to shrink progressively in subsequent observations, further confirming the secondary therapeutic effect of PDT (**Figures 9C, H**). Sparse LED-PDT induced apoptosis with secondary killing effects (**Figure 9H**). On day 3, apoptosis mostly occurred in the superficial layer of the tumor. On day 7, apoptosis uniformly occurred in the deep part of the tumor, with a significant increase in apoptotic cells (32.69%). On day 14, apoptosis was not observed, and tumor tissue recurred while shrinking. Point LED-PDT induced apoptosis around the necrotic tumor, and the secondary PDT effect also expanded around the necrotic tissue (**Figures 9C, H**).

Tumor recurrence was observed 8 days after both sparse LED-PDT and point LED-PDT (**Figure 8B**), which was confirmed with Ki-67 staining (**Figure S9**). Tumor recurrence was not observed until day 8 because secondary damage caused by PDT played a role in the early stage. The positive tumor cells in the tissue irradiated with the sparse LED array increased from 16.96% on day 7 to 58.35% on day 14, as shown in **Figure S9(B)**. This was also confirmed by the high expression of VCAM-1 and ICAM-1 in tumor tissues subjected to sparse LED-PDT, especially on day 14 (**Figures 9I, J**). After point LED-PDT, tumor recurrence was limited to the deep unirradiated residual tumor tissue, and angiogenesis mostly occurred in this area.

## 4 Discussion

Because of the development of LEDs, specifically implantable LEDs, in recent years, PDT has gained broader application prospects. Existing research mainly evaluated the performance of PDT mediated by LEDs by systematically adjusting the wavelength, power density, and energy density. Notably, the results indicated that LEDs can replace lasers. However, when an LED array is required to cover a large spot, the light field must be fully considered. In this study, we constructed three LED arrays: an intensive LED to produce a uniform light field, a sparse LED array to produce a non-uniform light field, and a point LED array to produce a Gaussian-like light field. We evaluated the performance of PDT mediated by these arrays in treating GI cancer *in vitro* and *in vivo* and investigated the mechanism of cell death for each case.

PDT is highly dependent on the availability of oxygen (34, 35). Therefore, oxygen distribution in cells and tissues should be fully considered in the design of the light source and the choice of photosensitizer. We considered the relationship between oxygen consumption and LED light field in the design of the above LED arrays. We found that the efficiency of PDT mediated by the intensive LED and point LED arrays was higher than that mediated by the sparse LED array both *in vitro* (**Figure 2**) and *in vivo* (**Figure 8**). The optical power density we used for the comparative analysis was low (1.25–2.5 mW/cm^2^ *in vitro* and 20 mW/cm^2^ *in vivo*), in the range of metronomic photodynamic therapy (mPDT) (36, 37). In this range, oxygen consumption is lower than oxygen supply, and oxygen is no longer a decisive factor for PDT efficiency. However, the effective optical power (minimum optical power that could effectively excite the photosensitizer) plays a decisive role in this case. For the sparse LED array, the proportion of regions receiving less than the effective optical power was large, resulting in the low PDT efficiency. In animal experiments (**Figure 9**; **Figure S5**), tumor recurrence was observed within the effective light penetration depth after sparse LED-PDT owing to insufficient effective light power density. However, after point LED-PDT, tumor recurrence was observed only in areas outside the effective illumination area. In contrast to these two cases, the tumor was completely suppressed by intensive LED-PDT because the effective light power density was supplied to the whole irradiation area and tumor depth. Notably, only 0.027 ± 0.004 cm^3^ of the tumor remained on day 14, which was considered complete disappearance of the tumor (**Figure 8B**).

With mPDT, light is delivered continuously at low rates for extended periods of time, and oxygen can react sufficiently with the active photosensitizing drug to destroy the target tumor by inducing apoptosis (36, 38, 39). Our study showed that mPDT mediated by different LED arrays could induce apoptosis, although the apoptosis rate and apoptosis pathway were different. Intensive LED-PDT induced the highest rate of apoptosis (**Figure 3A**) than sparse LED-PDT and point LED-PDT. Furthermore, proteomic results showed that the DEPs induced by intensive LED-PDT were related to mitochondrial activities, while those induced by sparse LED-PDT and point LED-PDT were mainly involved in extracellular and intracellular activities (**Figures 6**, **7**). Moreover, PDT mediated by all three LED arrays induced DNA repair and cellular metabolic activities (**Figure 6**), indicating that tumor cells underwent a self-repair process after PDT and thus confirming the involvement of apoptosis and autophagy (40–42).

The efficacy of PDT is related to the combined action of photosensitizer, light, and oxygen (43), and the performance of the last two agents is related to the light field. However, there is a threshold: the effective optical power density. In this study, there were regions in the non-uniform light field (sparse LED array) that were below the threshold, and because these regions were not effectively irradiated, tumor recurrence was inevitable. The influence of the three light fields on PDT when oxygen consumption is greater than oxygen supply, above the threshold, will be tested in our subsequent study. In practical applications, if a uniform light field is technically difficult to achieve, a non-uniform light field may be a better choice than a Gaussian-like light field because of its larger penetration depth. However, attention should be paid to ensure that the minimum power density is required for achieving effective tumor inhibition.

## 5 Conclusions

We studied the effect of different light fields on the performance of PDT for GI cancer *in vitro* and *in vivo* and investigated the mechanism of cell death for each case. From our preliminary data, we can conclude that death of GI cancer cells is more severe and occurs earlier with intensive LED-PDT than with sparse LED-PDT and point LED-PDT at the same dose with low power densities (1.25–2.5 mW/cm^2^ *in vitro* and 20 mW/cm^2^ *in vivo*), in which apoptosis mediated by mitochondria pathway played an important role. While the upregulation of ribosomal proteins and high expression of VCAM-1 and ICAM-1 after sparse LEDs-PDT inhibited the killing effect. On the basis of the results obtained in this study, we will develop micro-light sources for the digestive tract, to replace endoscopy for PDT of digestive tract tumors, thus reducing side effects and increasing PDT efficacy.

## Data availability statement

The original contributions presented in the study are included in the article/**Supplementary Material**. Further inquiries can be directed to the corresponding author.

## Ethics statement

The animal study was reviewed and approved by Institute of Radiation Medicine(CAMS)—Animal Ethics Committee.

## Author contributions

HY brought forward the subject and guided the writing. XS was major contributor in conducting the experiments and writing the manuscript. XD and YL designed and manufactured the light sources. HL was responsible for data processing. All authors contributed to the article and approved the submitted version.
