# Supplementary material for: Photodynamic therapy with light-emitting diode arrays producing different light fields induces apoptosis and necrosis in gastrointestinal cancer
Source: Front Oncol. 2022 Dec 16;12:1062666. doi: 10.3389/fonc.2022.1062666 (PMC9801516; doi:10.3389/fonc.2022.1062666)
Supplement: Supplementary file 1 [file DataSheet_1.doc]

Supplementary Material

**Photodynamic therapy with light-emitting diode arrays producing different light fields induces apoptosis and necrosis in gastrointestinal cancer**

Xiafei Shi^1,2^，Huijuan Yin^1,^*，Xiaoxi Dong^1^，Hongxiao Li^1^，Yingxin Li^1^

^1^Laboratory of Laser Medicine, Institute of Biomedical Engineering, Chinese Academy of Medical Sciences, Peking Union Medical College, Tianjin 300192, China

^2^School of Life Sciences, Tiangong University, Tianjin 300387, China

*** Correspondence:** Huijuan Yin
yinzi490@163.com

# Materials and methods

The coherent light field from the point LED array was classified into the Gaussian beam, flat-top Gaussian beam, and flat-top Gaussian beam of central depression according to the difference in the equivalent point light source spacing l, as shown in the Figure S1. In this study, the D86 beam width definition and light field uniformity index (modulation degree M) were jointly applied, and the variation of M as l increased was extracted. The light field corresponding to the Gaussian beam and flat-top Gaussian beam was accurately calculated. The range of l corresponding to the Gaussian beam, flat-top Gaussian beam, and flat-top Gaussian beam of central depression was accurately calculated.

The spot radius r was defined where the light intensity of an independent point light source was attenuated to 1/e of the central light intensity. The light field presented a Gaussian distribution when l < r, a flat-top Gaussian distribution when r ≤ l < 2r, a flat-topped Gaussian distribution of central depression when 2r ≤ l < 4r, and an independent Gaussian distribution again when l ≥ 4r.

In this experiment, we defined a flat-top Gaussian distribution (r ≤ l < 2r) as a uniform light field (Figure S1(B)), a flat-topped Gaussian distribution (2r ≤ l <4r) of central depression as a non-uniform light field (Figure S1(C)), and an independent Gaussian distribution (l ≥ 4r) as a Gaussian-like light field (Figure S1(D)).


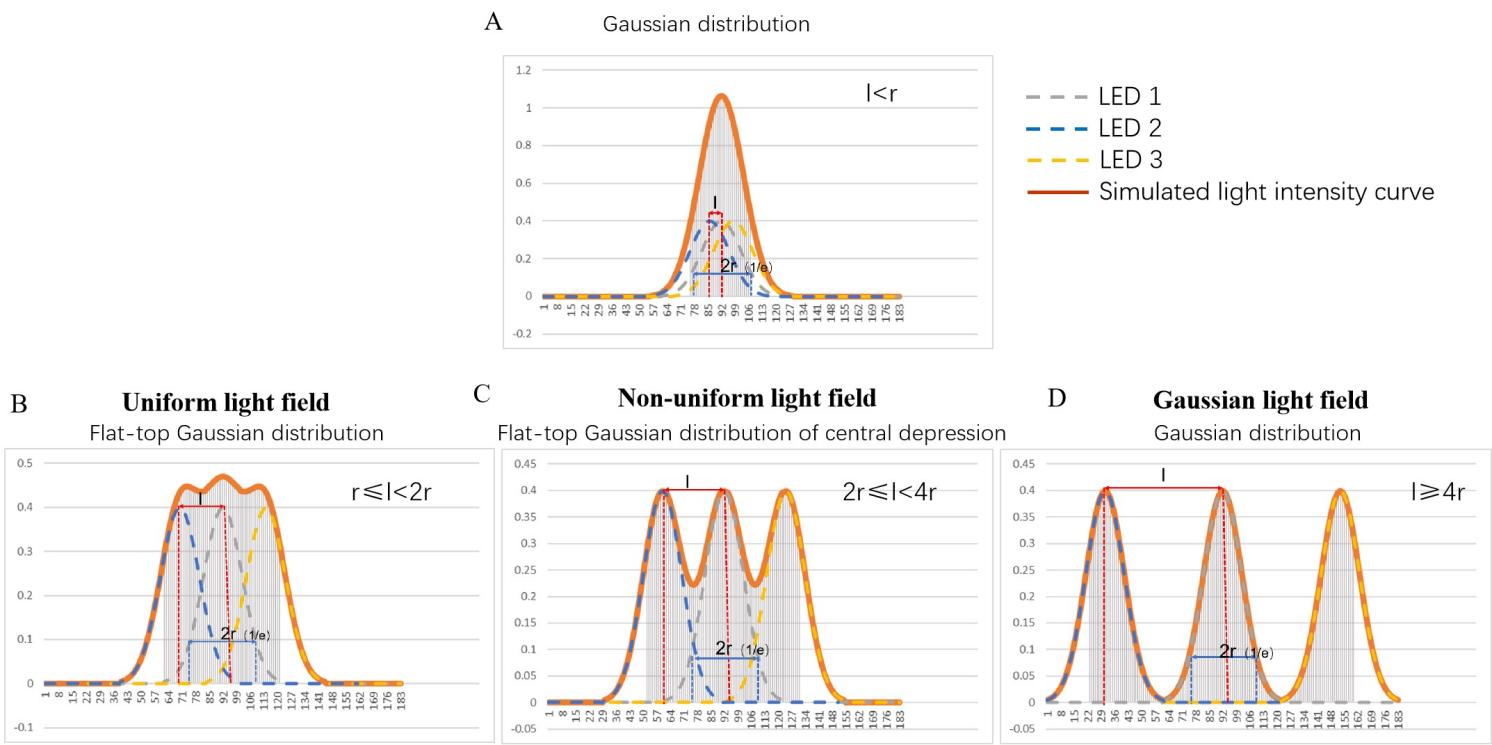


**Figure S1**. Definition of three light fields from algorithmic analyses: **A** Gaussian distribution, **B** uniform light field, **C** non-uniform light field, and **D** Gaussian-like light field.

For *in vitro* cell experiments, a module corresponding to a 6-well plate was designed for the different LED arrays, as shown in Figure S2(A). The module consisted of 208 LEDs, 16 LEDs, and 1 LED for the intensive LED array, sparse LED array, and point LED array, respectively. The circuit was set up to have a high-power USB adjustable power supply (15 W, 0.5–30 V) on each channel to allow independent voltage control for each set of LEDs. To accurately assess light delivery during *in vitro* cell culture irradiation, a 3D-printed black light barrier matching the 6-well plate was placed between the LED device and 6-well plate. A representative beam profile of each LED module was recorded using a beam quality analyzer (Ocean Optics, UK). Subsequently, each LED device was assessed to ensure a stable irradiance output over specific exposure times using a power density meter. Light sources used for *in vivo* animal experiments were similarly constructed, as shown in Figure S2(B).


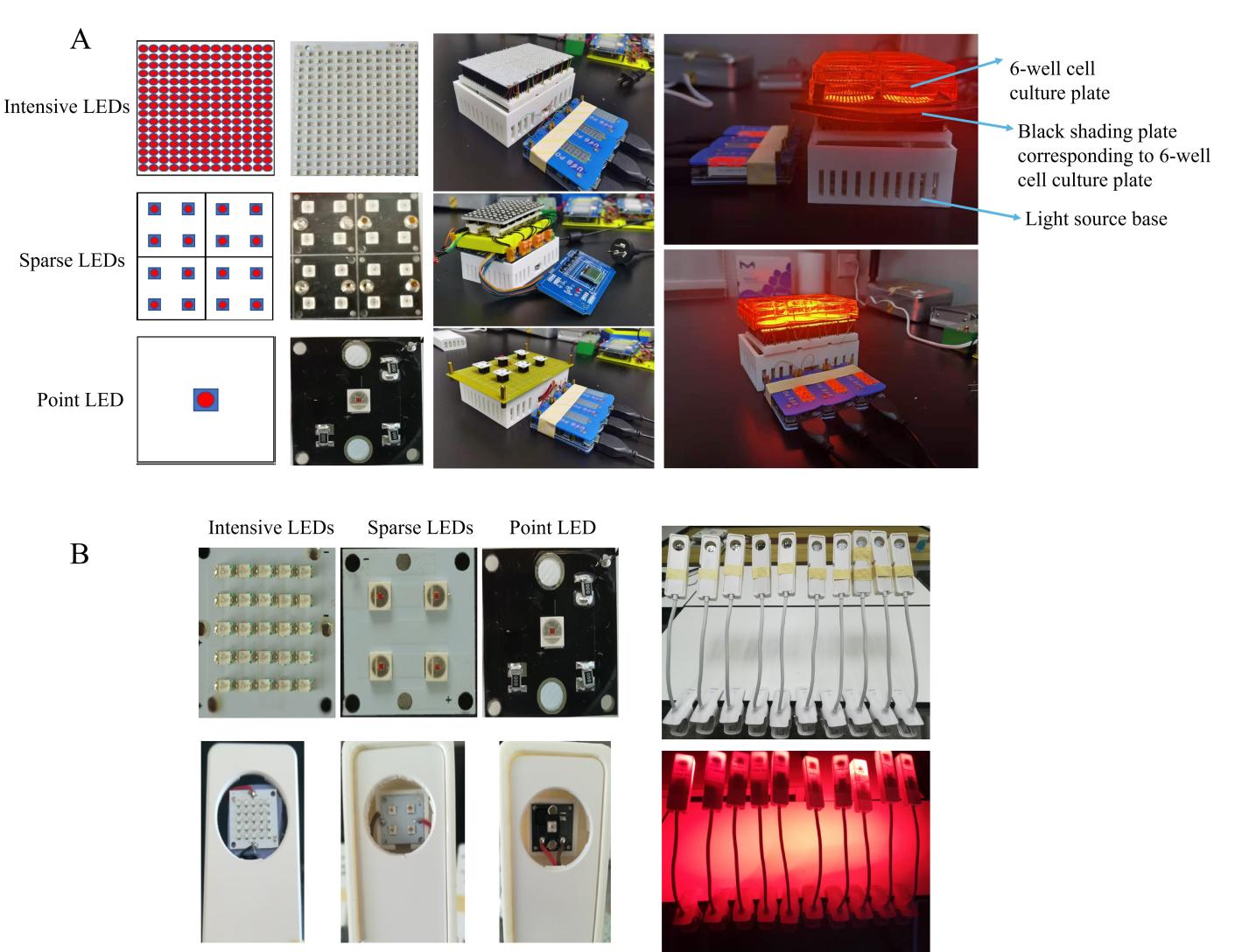


**Figure S2**. Three LED arrays with different light fields for *in vitro* cell experiments(**A)** and *in vivo* animal experiments**(B)**. Intensive LED array produces a uniform light field, sparse LED array produces a non-uniform light field, and point LED array produces a Gaussian-like light field.

Notably, for all three LED arrays, a specific heat dissipation design using silica gel sheets with good thermal conductivity was made, as shown in Figure S3(A). And the thermal damage of LED to cells was avoided owing to rapid and effective heat dissipation during the experiment, as shown in Figure S3(B), the cell viability was above 90% when exposed only to light from LED array at different energy density (0, 3, 6, 12 and 24 J/cm^2^).


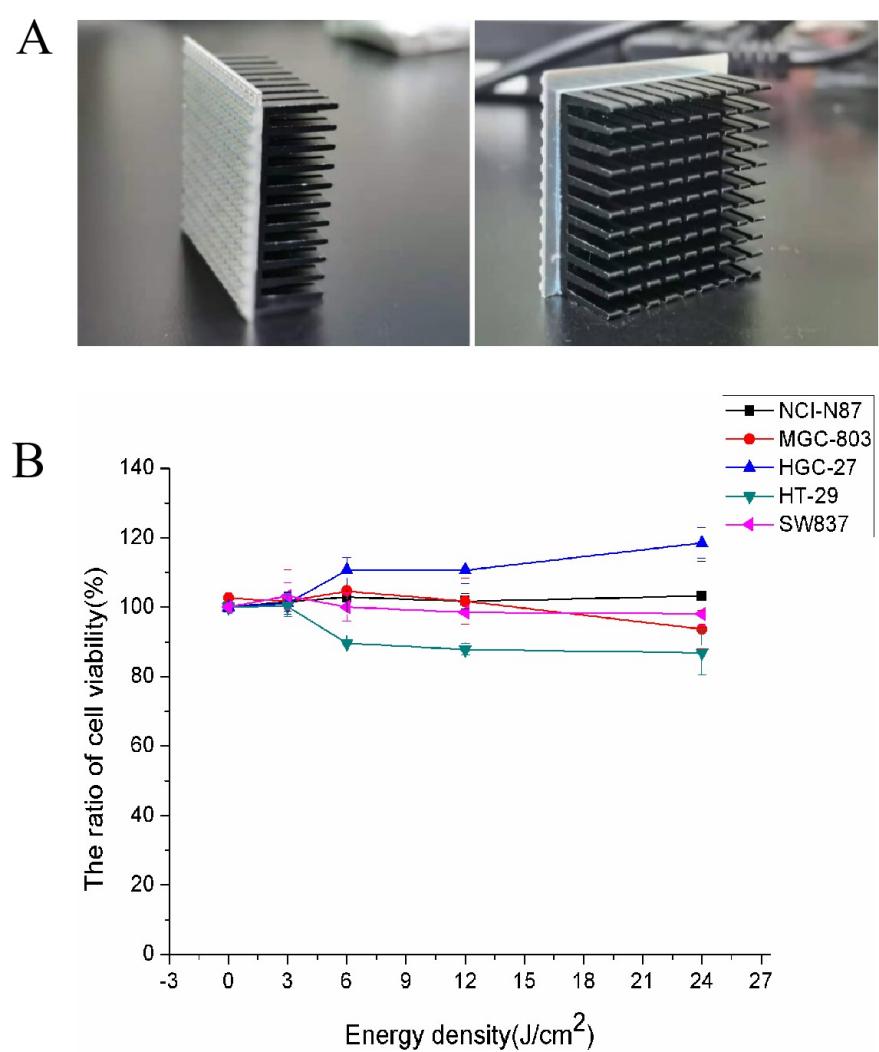


**Figure S3.** Heat dissipation design of LED arrays**(A)** and thermal damage to different cell lines(B).


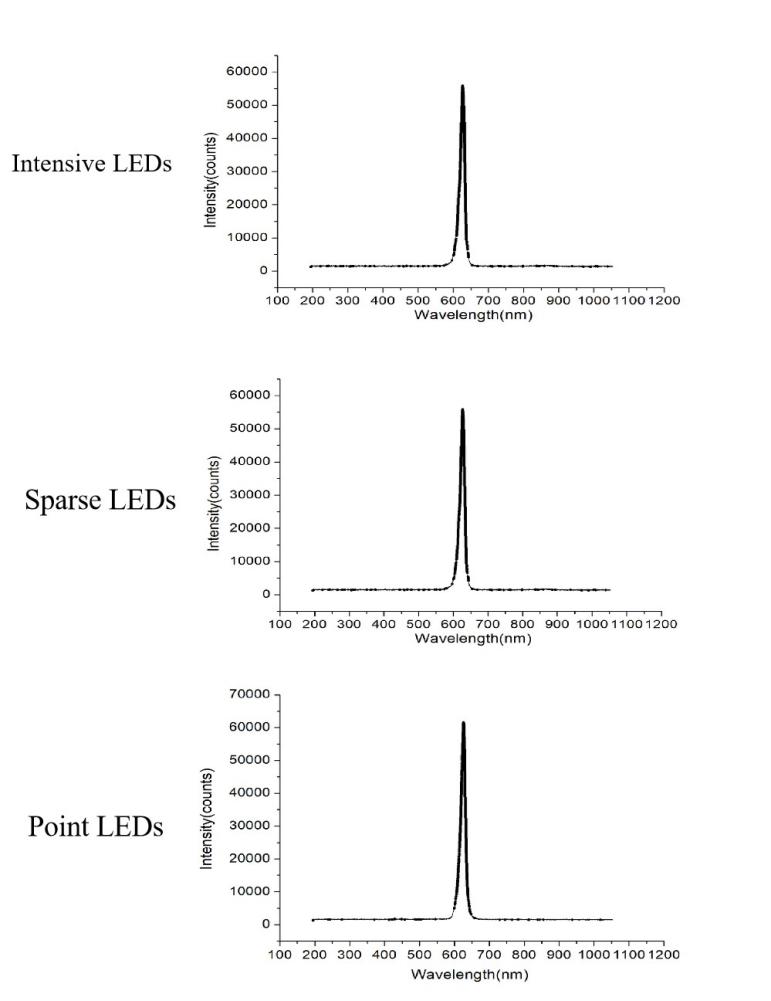


**Figure S4**. Spectrum of three LED arrays with different light fields. **Top**: Intensive LED array produces a uniform light field. **Middle**: Sparse LED array produces a non-uniform light field. **Bottom**: Point LED array produces a Gaussian light field.

# Results

For Monte Carlo stimulations, the tissue model, comprising dermis (thickness of 0.12 mm), tumor, and fat tissues, was approximated as a sphere with a diameter of 1 cm. The optical properties[1,2] of each tissue are presented in Table S1. The Monte Carlo method, along with a 3D-voxelized simulation tool (MCVM[3]) for heterogeneous media, was used to simulate the distributions of absorbed energy and flux. The tissue was divided into 0.02-mm^3^ voxels, and the incident point was determined according to the light source: intensive LED array, sparse LED array, or point LED array. We employed 1 × 10^6^ photon packages to simulate the distribution. The size of the absorption matrix was the same as that of the tissue model. The fluence distribution was calculated using the absorption matrix and published absorption coefficients[4].

**Table S1** Optical properties of tissues at 635 nm

| Tissue type | $\mu$_a_ (cm^-1^) | $\mu$_s_ (cm^-1^) | g | n |
| --- | --- | --- | --- | --- |
| Dermis | 0.1 | 187 | 0.81 | 1.37 |
| Fat | 0.67 | 118 | 0.9 | 1.40 |
| Tumor | 0.49 | 270 | 0.97 | 1.43 |

MCVM simulation results showed that the distribution of light along the X and Y axes of the tumor was the same. Therefore, the distribution of light in the tumor was represented by that along the X-axis, as shown in Figure S5. The light output fitting results for the intensive LED, sparse LED, and point LED arrays are shown from top to bottom. After irradiation with the intensive LED array or point LED array, the distribution of light in the tumor showed a single exponential decline, and the decrease with the point LED array was fast. For example, after irradiation with the intensive LED array, light absorption and luminous flux tended to be 0 at the tissue depth of 6000 mm. However, the transmission distance of light from the point LED array was only 4000 mm. After irradiation with the sparse LED array, light absorption and luminous flux first increased exponentially as the tissue depth increased from 0 to 920 m (0 < X < 920 m) and then decreased exponentially as the depth further increased beyond 920 m (X > 920 m).

Overall, in tissue, the transmission distance of light from the sparse LED and intensive LED arrays was the same, reaching a depth of 6000 mm, which was significantly deeper than that of light from the point LED array (4000 mm). Moreover, at each tissue depth, the absorption and luminous flux of light from the sparse LED array were the lowest among light from the three LED arrays.


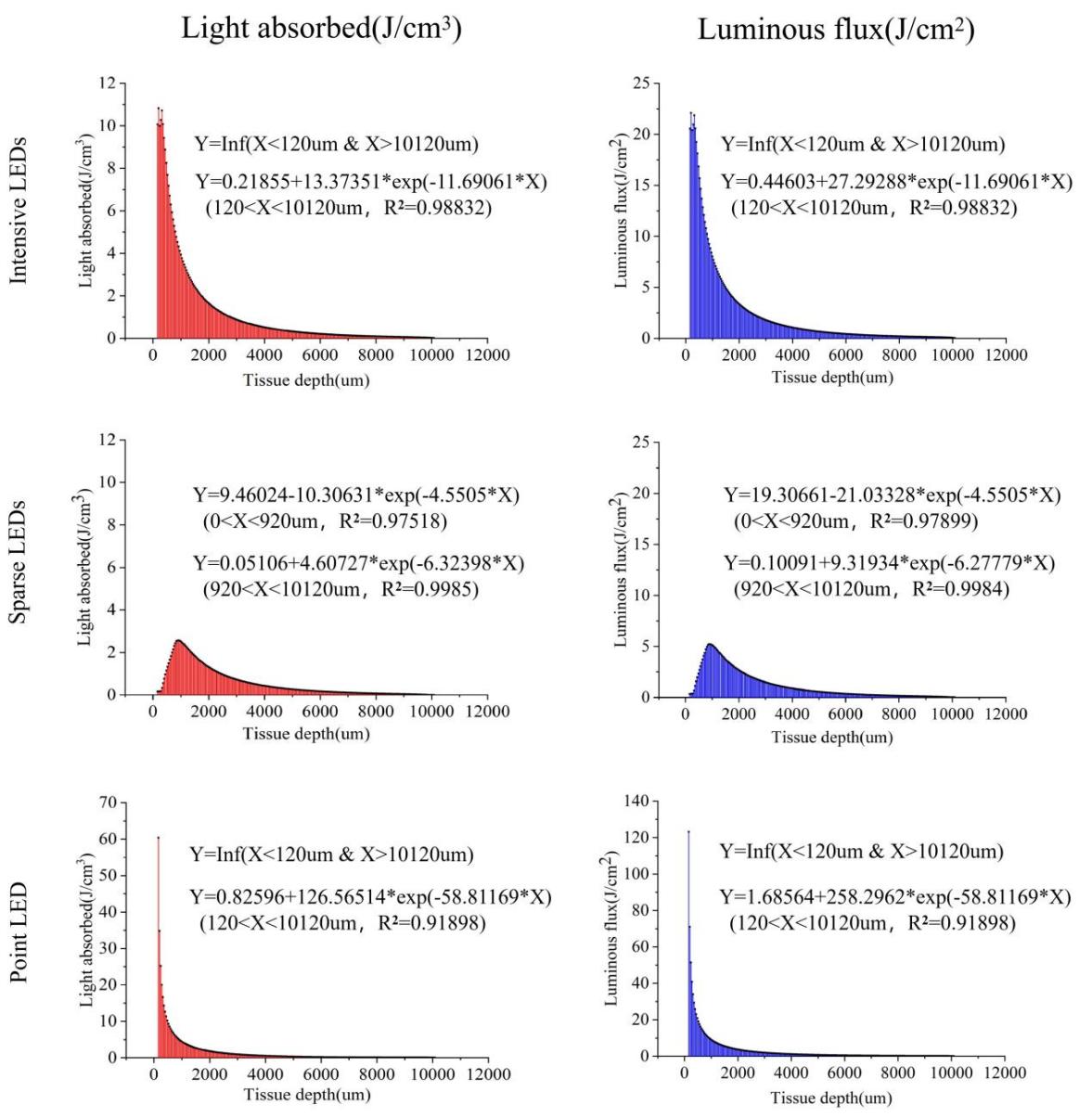


**Figure S5**. Monte Carlo simulation of the distribution of light from three light sources producing different light fields for *in vitro* tumor cell experiments. **Top**: Intensive LED array produces a uniform light field. **Middle**: Sparse LED array produces a non-uniform light field. **Bottom**: Point LED array produces a Gaussian light field.

We first analyzed the effect of LED-PDT on five kinds of GI cancer cells (NCI-N87, MGC-803, HGC-27, SW837 and HT-29) under different HpD concentrations (3.125, 6.25, 12.5, 25, and 50 μg/mL), energy densities (0, 3, 6, 12 and 24 J/cm^2^), and power densities (1.25, 2.5, 5, 10, and 20 mW/cm^2^) and at different times after PDT (2, 12, and 24 h). As shown in Figure S6, different GI cancer cell lines have different tolerances to the above parameters. From these results, the optimal PDT parameters (minimum dose with cell mortality greater than 70%) for each cell line were determined, as shown in Table S2.


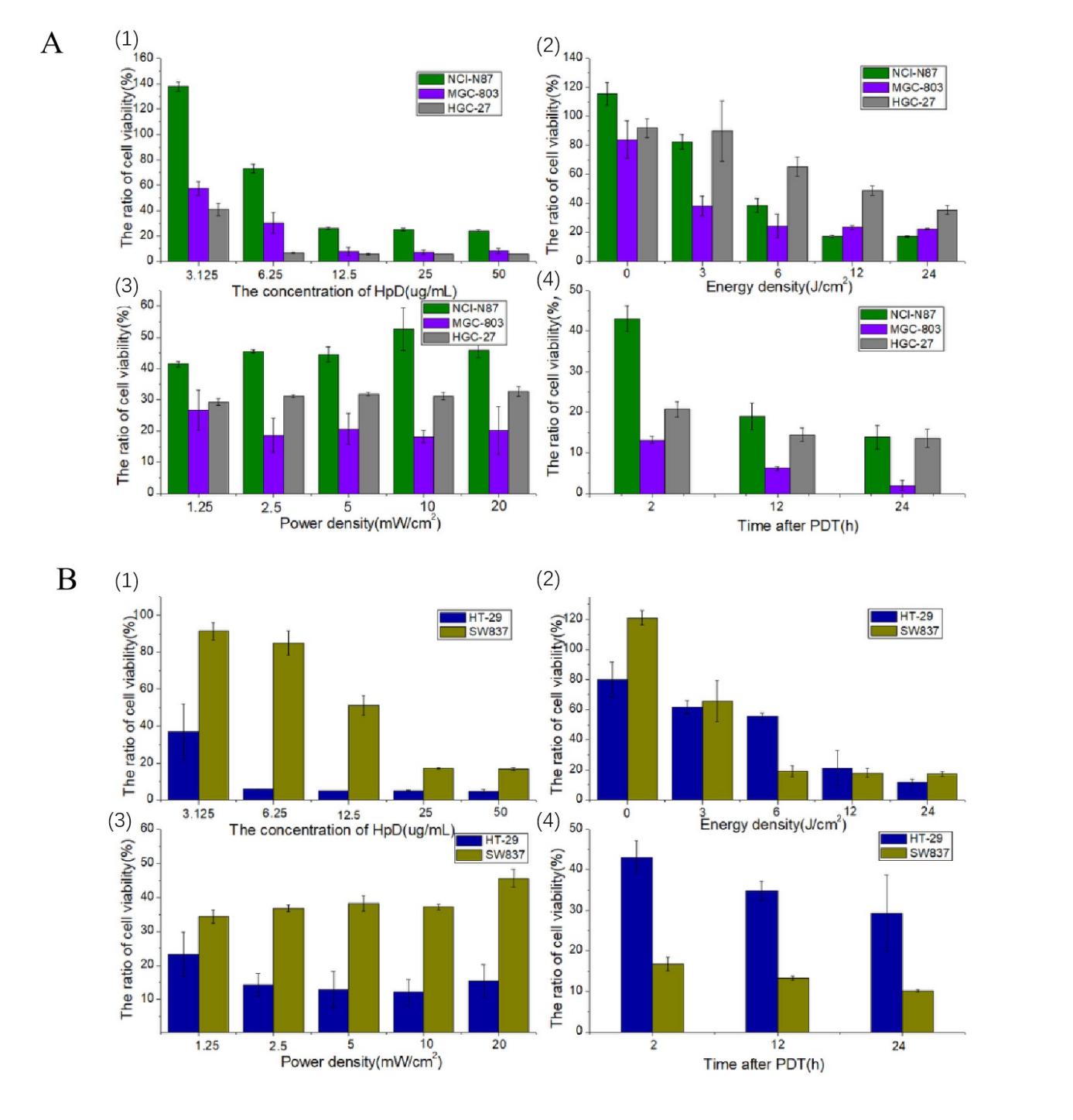


**Figure S6**. Effect of LED-PDT on five kinds of GI cancer cells. **A** (1)–(4) Effect of LED-PDT on gastric cancer cells NCI-N87 (highly differentiated), MGC-803 (poorly differentiated), and HGC-27 (undifferentiated) under (1) different HpD concentrations, (2) different energy densities, (3) and different power densities and at (4) different times after PDT. **B** (1)–(4) Effect of LED-PDT on colon cancer cell HT-29 and rectal cancer cell SW837 under (1) different HpD concentrations, (2) different energy densities, and (3) different power densities and at (4) different times after PDT.

**Table S2**. Optimal PDT parameters for different GI cancer cells

| Cell lines | HpD (μg/mL) | Energy density (J/cm^2^) | Power density  (mW/cm^2^) | Irradiation time (min) | Time after PDT (h) |
| --- | --- | --- | --- | --- | --- |
| NCI-N87 | 12.5 | 12 | 1.25 | 160 | 12 |
| MGC-803 | 12.5 | 6 | 2.5 | 40 | 2 |
| HGC-27 | 6.25 | 6 | 1.25 | 80 | 2 |
| HT-29 | 6.25 | 12 | 2.5 | 80 | 24 |
| SW837 | 25 | 6 | 1.25 | 80 | 2 |


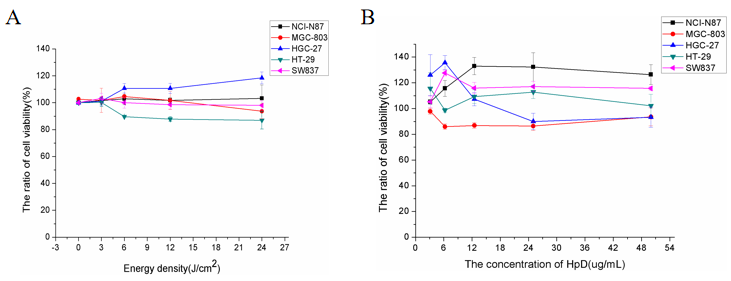


**Figure S7** Detection of LED phototoxicity**(A)** and HpD dark toxicity**(B)** in each cell lines. **A** Light and no HpD. **B** HpD and no light.


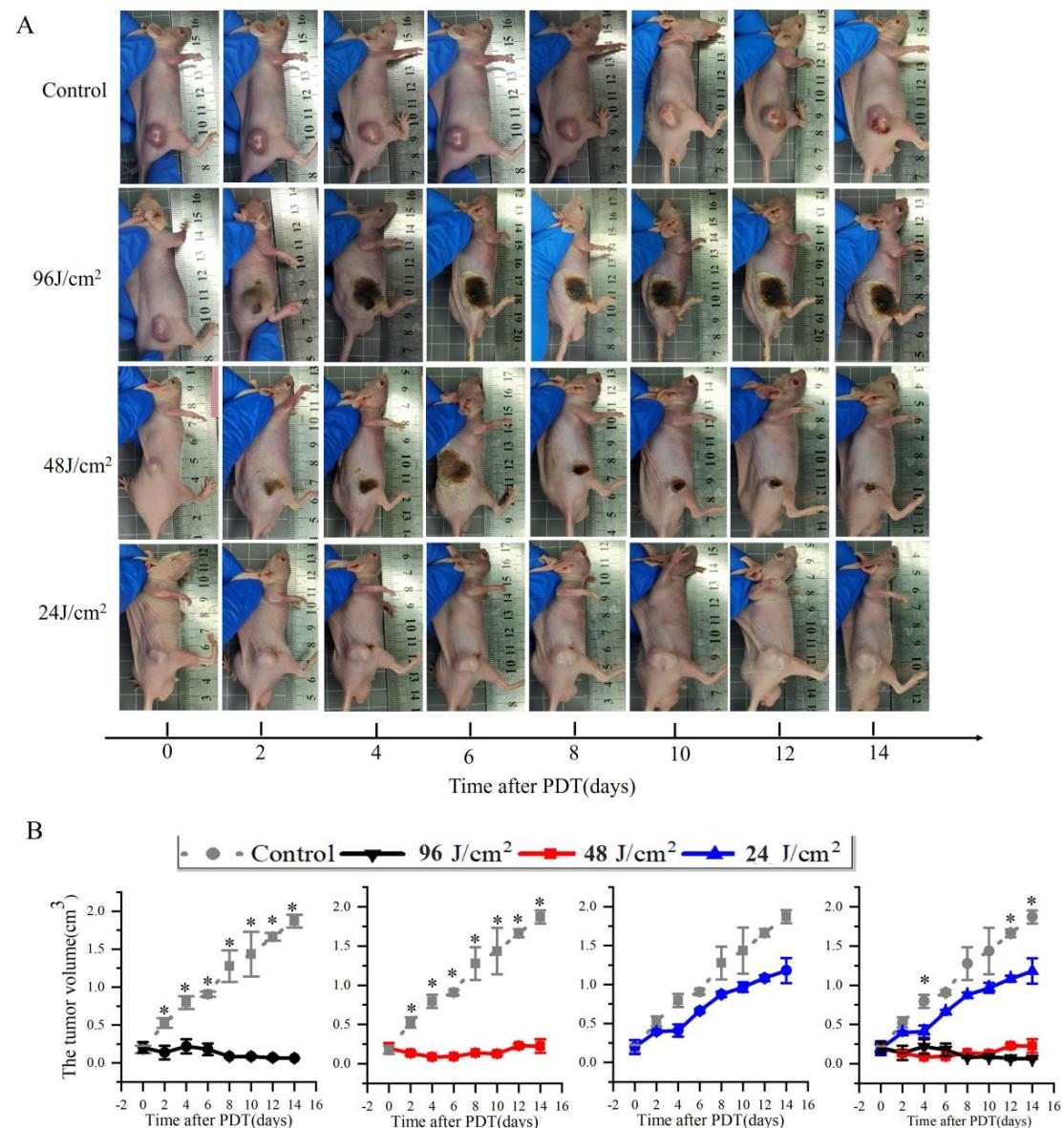


**Figure S8**. Effect of LED-PDT on tumor-bearing nude mice under different light energy density conditions (96, 48, and 24 J/cm^2^). **A** Tumor morphology photographed every two days after PDT. **B** Changes in tumor volume with time after PDT. *P<0.05 by one-way ANOVA. Mean ± SD in broken line graphs.


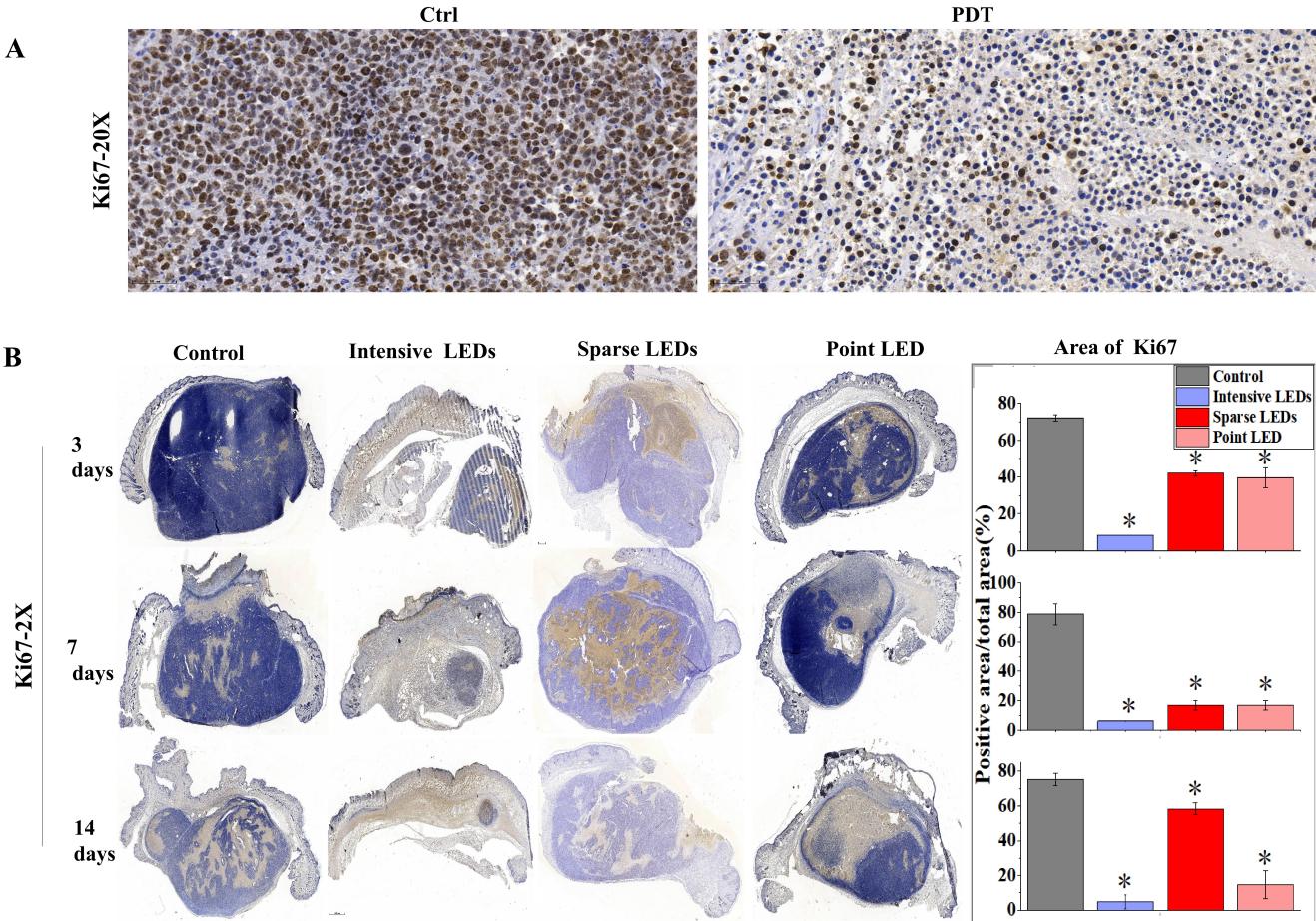


**Figure S****9**. MGC-803 tumor cell proliferation after PDT mediated by different light fields. MGC-803 tumor-bearing mice were sacrificed on days 3, 7, and 14 after PDT. **A** Tumor tissue sections were stained with Ki67 and then scanned with a 40× microscope and magnified 20×. **B** Coronal section scans of tumor tissue stained with Ki67 and quantitative analysis of positive area. (Mean ± SD, *P<0.05 by one-way ANOVA compared with control group.)
